# Black Phosphorus Field-Effect Transistors with Improved Contact via Localized Joule Heating

**DOI:** 10.3390/nano13182607

**Published:** 2023-09-21

**Authors:** Fangyuan Shi, Shengguang Gao, Qichao Li, Yanming Zhang, Teng Zhang, Zhiyan He, Kunchan Wang, Xiaowo Ye, Jichao Liu, Shenghao Jiang, Changxin Chen

**Affiliations:** National Key Laboratory of Advanced Micro and Nano Manufacture Technology, Key Laboratory for Thin Film and Microfabrication of Ministry of Education, School of Electronic Information and Electrical Engineering, Shanghai Jiao Tong University, Shanghai 200240, China

**Keywords:** black phosphorus (BP), contact resistance, field-effect transistors (FETs), Joule heating, two-dimensional (2D) material

## Abstract

Two-dimensional (2D) black phosphorus (BP) is considered an ideal building block for field-effect transistors (FETs) owing to its unique structure and intriguing properties. To achieve high-performance BP-FETs, it is essential to establish a reliable and low-resistance contact between the BP and the electrodes. In this study, we employed a localized Joule heating method to improve the contact between the 2D BP and gold electrodes, resulting in enhanced BP-FET performance. Upon applying a sufficiently large source–drain voltage, the zero-bias conductance of the device increased by approximately five orders of magnitude, and the linearity of the current–voltage curves was also enhanced. This contact improvement can be attributed to the formation of gold phosphide at the interface of the BP and the gold electrodes owing to current-generated localized Joule heat. The fabricated BP-FET demonstrated a high on/off ratio of 4850 and an on-state conductance per unit channel width of 1.25 μS μm^−1^, significantly surpassing those of the BP-FETs without electrical annealing. These findings offer a method to achieve a low-resistance BP/metal contact for developing high-performance BP-based electronic devices.

## 1. Introduction

Two-dimensional (2D) black phosphorus (BP) exhibits an adjustable thickness-dependent bandgap [1,2], high carrier mobility [3,4], and excellent mechanical properties [5,6], rendering it an excellent candidate for field-effect transistors (FETs). However, the BP-FET contact significantly influences device characteristics, such as the on-state current and on/off current ratio. The interface state between the 2D BP and a metal electrode plays a critical role in their contact. Typically, when 2D BP contacts the metal electrode via the van der Waals (vdW) interaction force, a relatively large barrier height or width and a Fermi level pinning effect exists between the 2D BP and the metal electrode, which can result in a large contact resistance and thereby degrade the device performance. Thus, establishing a reliable and low-resistance contact between BP and metal electrodes is crucial for achieving high-performance devices.

Some methods have been proposed to reduce contact resistance previously. Thermal annealing could improve the contact by desorbing residues and absorbates on BP [7,8]. However, the annealing temperature was limited such that the metal-catalytic amorphization or sublimation of the BP would occur at about 300 °C [7,9]. Selecting metals with suitable work functions to form a small Schottky barrier with BP was another approach to reducing the contact resistance [10,11,12,13,14,15]. For example, using Ni or NiCr alloy as the contact metal has been reported to achieve a relatively low contact resistance of 6.3 kΩ μm and 18.1 kΩ μm for the BP-FETs, respectively [14]. However, the obtained contact resistance was still relatively large due to the side-contact nature between the metal and the BP. Doping BP with lithium has been employed as a method to reduce the contact resistance, creating a narrower carrier injection barrier at the contact [16]. Nonetheless, this method introduced contamination and instability. Thus, a reliable and low-resistance contact method is highly desired.

To address this, we developed a facile method using localized Joule heating (electrical annealing) to improve the contact and fabricate a high-performance BP-FET. By using this approach, the zero-bias conductance (G_0_) of the device was significantly increased by five orders of magnitude. Electrical annealing enabled the formation of an ohmic contact between the BP and Au electrodes, resulting in improved linearity in the current–voltage output characteristics. The fabricated BP-FET demonstrated a high on/off ratio of 4850 and an on-state conductance (G_on_) per unit channel width of 1.25 μS μm^−1^, significantly surpassing those of the BP-FETs without electrical annealing.

## 2. Material and Methods

### 2.1. Preparation of the 2D BP on the PDMS or Si Substrate by Using the Mechanical Exfoliation Method

Using the tape-assisted mechanical exfoliation method [17], the bulk BP crystals could be exfoliated into thin 2D BP sheets. The tape with 2D BP sheets was subsequently pressed onto a 5 mm × 5 mm polydimethylsiloxane (PDMS) substrate with moderate finger pressure at room temperature (~25 °C) for 10 s. The tape and the PDMS were separated to obtain the sample with 2D BP on the PDMS substrate. 

To prepare 2D BP on the SiO_2_/Si substrate, the PDMS substrate with the 2D BP was pressed onto the SiO_2_/Si substrate for 20 s. The sample was heated at 45 °C for 5 min before separating the PDMS and the SiO_2_/ Si substrate. The resulting sample was soaked in acetone to remove the residue of the PDMS and then blown dry.

### 2.2. Atomic Force Microscopy (AFM) Characterization of 2D BP and BP-FETs

The AFM measurements of the samples were conducted using a Dimension Icon and Fast-Scan Bio AFM from Bruker, Germany. The commercial AFM tips (Model: OMCL-AC240TS-R3, Olympus Micro Cantilevers Company, Tokyo, Japan) were utilized in the measurements. These AFM tips have the following specifications: tip radius of 7 nm, spring constant of 1.7 N m^−1^, and resonant frequency of 70 kHz. 

### 2.3. Fabrication of the 2D BP-Based FETs

We fabricated a BP-FET with a 2D BP sheet on Au electrodes. The Au (50 nm)/Cr (2 nm) source and drain electrodes with a gap of 365 nm were fabricated via electron-beam lithography (EBL) and lift-off on a Si substrate with a 100 nm-thick thermally oxidized SiO_2_ layer. The 2D BP on the PDMS was subsequently transferred onto the metal electrodes as the device channel via a dry transfer method in an alignment-transferring platform. Firstly, high-quality 2D BP with a width of 4 μm on the PDMS substrate was selected and placed onto the metal source and drain electrodes via an alignment transfer instrument. Then, the sample was heated at 45 °C for 5 min followed by the separation of the PDMS and SiO_2_. The resulting sample was soaked in acetone for 30 min to remove the remaining PDMS on the surface. In this way, the 2D BP was transferred to the SiO_2_/Si substrate with its two ends on the metal source and the drain electrodes. The length and width of the 2D BP channel were 365 nm and 4.0 μm, respectively. The Si and SiO_2_ layers in the device were used as the gate and gate insulator of the FET, respectively.

We also fabricated a BP-FET with a 2D BP sheet underneath the Au electrodes. A 2D BP sheet with the same width of 4 μm on the PDMS substrate was transferred to the Si wafers with a 100 nm-thick thermally oxidized SiO_2_ layer. Thereafter, the Au (50 nm)/Cr (2 nm) source and drain electrodes with a gap of 365 nm were fabricated on the 2D BP on the SiO_2_/Si substrate by the EBL and lift-off technique. The length and width of the 2D BP channel were also 365 nm and 4.0 μm, respectively. The Si and SiO_2_ layers in the device were used as the gate electrode and gate insulator of the FET, respectively.

### 2.4. Improvement of the 2D-BP/Au Contact by Using the Joule Heating Method

The gate voltage was not applied during the electrical annealing process. The electrical annealing was performed by gradually increasing the source–drain voltage (V_ds_) from 1 V to 16 V with a step size of 1 V. The applied V_ds_ generated Joule heat owing to the high resistance at the contact region, thereby increasing the local temperature and causing localized annealing at the contact interface. As a result, the electrical annealing significantly improved the contact in the device.

### 2.5. Electrical Measurements of the 2D BP-FETs

The electrical characteristics of the 2D BP-FET were tested using an Agilent B1500A semiconductor performance analyzer. The devices were tested in a vacuum of 8 × 10^−5^ Pa.

## 3. Results and Discussion

### 3.1. Preparation and Characterization of 2D BP-Based FET

**Figure 1:**
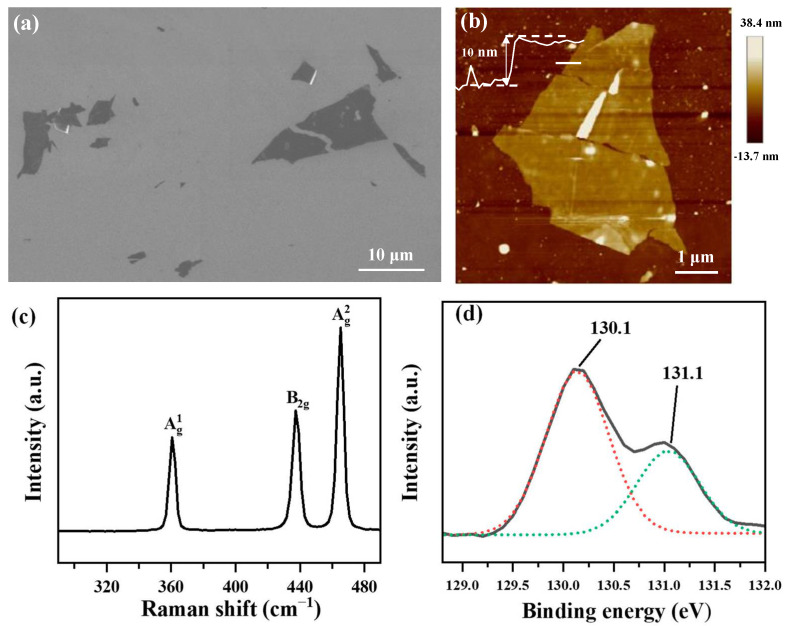
Images and Raman spectrum of the prepared 2D BP. (**a**) SEM image of the mechanically exfoliated 2D BP. (**b**) AFM image of an individual 2D BP. The height of the 2D BP is ~10 nm. (**c**) Raman spectrum and (**d**) XPS spectrum of the 2D BP. The red and green curves in (**d**) are the fitting curves of two XPS peaks.

First, high-quality bulk BP crystals were prepared using a short-way transport reaction [18,19], resulting in crystal branches with smooth surfaces and a metallic luster. The BP branches exhibited varying dimensions with widths ranging from 30 μm to 5 mm, lengths ranging from 3 mm to 10 mm, and thicknesses ranging from several micrometers to tens of micrometers. Subsequently, we prepared 2D BP by mechanically exfoliating the synthesized bulk BP crystals. The resulting 2D BP exhibited smooth surfaces with sizes ranging from a few micrometers to tens of micrometers (Figure 1a) and a typical thickness of approximately 10 nm (Figure 1b). The Raman spectrum of the 2D BP showed three characteristic peaks at 361, 437, and 465 cm^−1^, corresponding to the vibrational mode $A_{g}^{1}$, $B_{2g}$, and $A_{g}^{2}$, respectively (Figure 1c). A high $A_{g}^{1}/A_{g}^{2}$ ratio of 0.47 indicated no obvious oxidation in the 2D BP [20,21]. Figure 1d shows the P 2p spectra of the 2D BP. The bare BP shows P 2p doublet of P 2p_3/2_ and 2p_1/2_ at 130.1 and 131.1 eV, respectively, which can be assigned as P_0_ corresponding to the unreacted BP.

**Figure 2:**
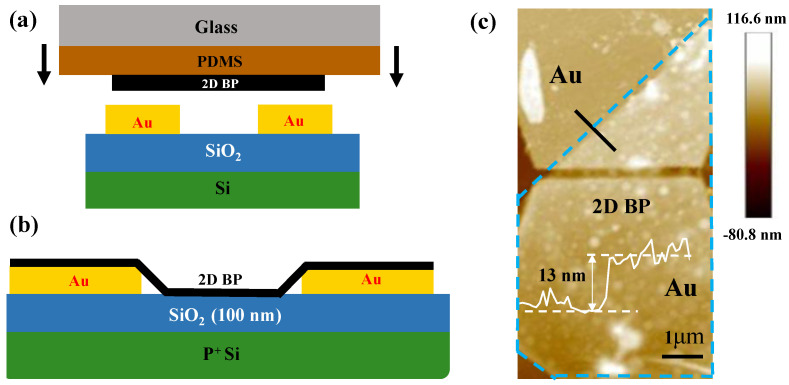
Process schematics and AFM image of the BP-FET. (**a**) Schematic of the transfer process of 2D BP to metal electrodes. (**b**) Schematic of the 2D-BP-based FET. (**c**) AFM image of the 2D-BP-based FET; Insert: height profile of the device with approximately 13 nm-thick 2D BP.

Subsequently, we fabricated a BP-FET with a 2D BP sheet on Au electrodes. During the experiment, the 2D BP was first pressed onto a PDMS substrate and subsequently transferred onto the prefabricated metal electrodes via a dry transfer method in an alignment-transferring platform to form a vertical BP-FET device, as shown in Figure 2a. For the detailed transfer processes, the reader can refer to the Material and Methods part. Figure 2b shows the schematic of the fabricated BP-FET. Figure 2c shows the AFM image of this device, exhibiting the 2D BP thickness of approximately 13 nm. 

### 3.2. Contact Improvement via Electrical Annealing

**Figure 3:**
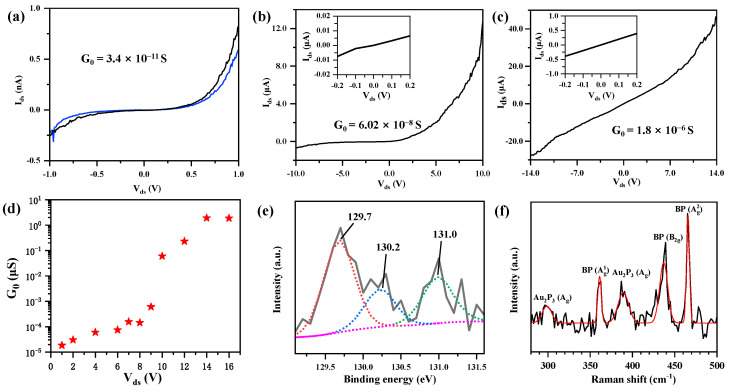
The I-V characteristics of the BP-FET in the electrical annealing process and the spectral characterizations at the BP/Au contact after the electrical annealing. (**a**–**c**) Typical I-V curves of the device at the increased sweeping V_ds_ of 1 V, 10 V, and 14 V with no gate voltage applied in the electrical annealing process. The blue and black curves in (**a**) are the I–V curves before and after the thermal annealing of 150 °C. Inset in (**b**,**c**): I–V curve near zero bias. (**d**) The G_0_ as a function of the applied V_ds_. (**e**) XPS and (**f**) Raman spectrum acquired from the contact region of the BP and Au after electrical annealing. In (**e**), the red, blue and green curves are the fitting curves of the three peaks; the purple line is the baseline. In (**f**), the red curve is the fitting curve of the peaks.

The impact of thermal and electrical annealing on the contact between the BP and the metal electrodes in the device was investigated. In the experiment, we attempted different thermal annealing temperatures up to 230 °C under a vacuum. The electrical curves of the device under different annealing temperatures were similar and no obvious improvement was observed after the thermal annealing. Figure 3a shows the I–V curve of a FET before (blue) and after (black) thermal annealing at 150 °C. No significant change of the G_0_ for the FET (V_g_ = 0 V) was observed after the thermal annealing. The G_0_ was calculated to be approximately 3.4 × 10^−11^ S. Subsequently, the electrical annealing was performed on the same device by gradually increasing the applied V_ds_ from 1 V to 16 V in steps of 1 V with the duration of the electrical sweeping (Joule heating) kept at 0.8 s. In the process of the electrical annealing, the Joule heat was generated owing to the high resistance at the contact, which increases the local temperature [22,23] and thereby results in localized annealing of the contact interface. As shown in Figure 3b, a significant increase in G_0_ was observed when the applied voltage reached 10 V with a calculated value of approximately 6.02 × 10^−8^ S, representing an increase of three orders of magnitude relative to the initial value. In Figure 3c, G_0_ reached 1.8 × 10^−6^ S when the applied voltage was increased to 14 V. Electrical annealing improved the contact in the device and increased the zero-bias conductance as the applied V_ds_ increased. In the experiment, we observed a change in the I–V curve from asymmetrical to symmetrical (Figure 3a–c) as the applied V_ds_ was increased to 13 V, and the I-V curves have a better linearity when the V_ds_ is larger than 13 V (Figure 3c). The asymmetrical I-V curves observed in Figure 3a,b were attributed to the inconsistent contact at the source and the drain when the applied V_ds_ was smaller than 10 V. The symmetrical I-V curve seen in Figure 3c occurred because the contact at the source and the drain became consistent when the applied V_ds_ was increased to 13 V. The details of this will be discussed later.

Figure 3d illustrates the G_0_ values at different V_ds_ values during the entire electrical annealing process. When the V_ds_ ranged from 1 V to 8 V, the increase in G_0_ was relatively slow, amounting to about an order of magnitude. However, at the V_ds_ of 8–14 V, a significant rise in G_0_ occurred, amounting to approximately four orders of magnitude. Beyond 14 V, G_0_ reached a state of saturated conductance, showing no further increase in the current when the applied V_ds_ was continually raised to 16 V. The electrical annealing process resulted in a substantial change in G_0_ from 3.4 × 10^−11^ to 1.8 × 10^−6^ S, indicating an increase of about five orders of magnitude with a rapid increase observed in the middle range of the process.

Compared with other reported methods for improving the contact of BP-FET, the Joule heating method led to a considerably larger reduction in resistance [7,15]. The Joule heating method was also used to improve the contact between other low-dimensional materials and metal [24,25,26]. 

### 3.3. Physical Mechanism of Electrical Annealing

To examine the reduction in resistance, we conducted X-ray photoemission spectroscopy (XPS) and Raman spectroscopy at the contact region. Figure 3e shows the XPS analysis with two peaks located at ~130.2 and ~131.0 eV, corresponding to the 2p_3/2_ and 2p_1/2_ binding energies of the P element in BP, respectively. The peak at 129.7 eV was assigned to the 2p_3/2_ peak of the P element in Au_2_P_3_ [27]. This observation was further confirmed by the Raman spectroscopy (Figure 3f), where three characteristic peaks at 362 cm^−1^, 439 cm^−1^, and 465 cm^−1^ correspond to BP and two peak positions (296 cm^−1^ and 387 cm^−1^) correspond to Au_2_P_3_ [28]. XPS and Raman characterization confirmed the generation of Au_2_P_3_ in the contact region.

The significant increase in the zero-bias conductance for 2D BP after electrical annealing can be explained as follows. When 2D BP sheets are placed on an Au electrode, the interface between the BP and Au electrodes exhibits a small vdW gap, acting as an additional tunneling barrier for carrier transport and thereby causing a large contact resistance. When a relatively small V_ds_ (<8 V) is applied during electrical annealing, the generated Joule heat causes the desorption and removal of residues and adsorbates, such as environmental molecules (e.g., water vapor and oxygen), resulting in a reduction in resistance. When a larger V_ds_ (8 V ≤ V_ds_ ≤ 14 V) is applied, a sufficiently large local Joule heat is generated at the interface between the BP and the Au electrode, leading to the formation of gold phosphide. Previous studies have shown that Au and P can form gold phosphide. Although Au can form compounds with red phosphorus [29] or indium phosphide [30] at temperatures above 400 °C, the Au_2_P_3_ compound was not observed in the thermal annealing process of the BP/Au contact at a limited temperature because BP can degenerate or decompose at temperatures above 300 °C. Theoretical calculations show that Au_2_P_3_ is a semiconductor with a narrow bandgap (0.16–0.22 eV) [27,31], which can serve as a semiconductor layer at the Au/BP interface, forming an ohmic contact with the metal and reducing the contact resistance. Previous reports have also shown that the formation of the metal phosphide at the contact of the other metals and the BP can also significantly reduce the contact resistance [32,33,34]. When the applied V_ds_ is larger than 14 V, no further rise of the current is observed because the BP and Au have reacted sufficiently before that.

### 3.4. Electrical Performance of 2D BP-FETs

**Figure 4:**
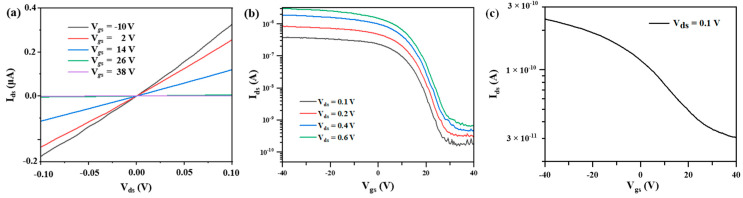
Electrical measurements of the BP-FET with a 2D BP sheet on the Au electrodes after and before electrical annealing. (**a**) Output and (**b**) transfer characteristic curves of the device after the thermal annealing at 150 °C followed by the electrical annealing. (**c**) Transfer characteristic curve of the device thermally annealed at 150 °C before the electrical annealing.

The performance of BP-FETs was studied. The drain–source current (I_ds_) curves of BP-FET after the Joule heating are shown in Figure 4a,b. As shown in Figure 4a, the I_ds_–V_ds_ curves are linear for −0.1 V < V_ds_ < 0.1 V, proving the formation of good contact. According to the transfer curves in Figure 4b, the device was modulated from the ON state to the OFF state with the increase of V_gs_, showing a p-type behavior. In contrast, the on/off ratio of the same device before the electrical annealing was about 8, as shown in Figure 4c. A previous study showed that the work function of the contact metal would affect the alignment of the Fermi level of the metal after the metal contacted the BP [35]. When the Au was used as the contact metal in our device, its Fermi level was aligned at the location close to the top of valence band, which resulted in a small hole Schottky barrier [36]. Thus, the device is in on-state at V_gs_ = 0 V, as shown in Figure 4b. The G_on_ normalized by the width of the BP channel was calculated to be 1.25 μS μm^−1^. The BP-FET displayed a high I_on_/I_off_ ratio of 4850 at V_ds_ = 0.6 V, which can be attributed to the electrical annealing that resulted in low contact resistance between the BP channel and the metal electrode.

**Figure 5:**
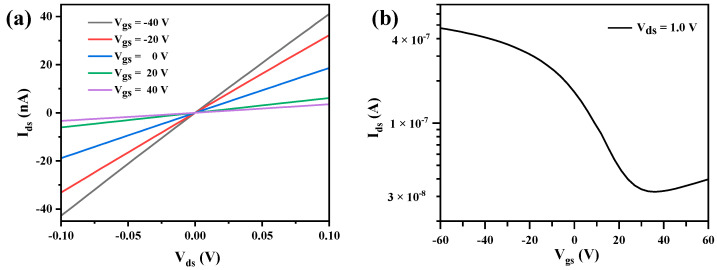
Electrical measurements of the BP-FET with a 2D BP sheet underneath the Au electrodes after thermal annealing. (**a**) Output characteristic curves of the device. (**b**) Transfer characteristic curves of the device measured at V_ds_ = 1 V.

Additionally, we fabricated a BP-FET with an identical channel length and width by depositing metal electrodes onto 2D BP with a similar thickness, followed by thermal annealing at 150 °C. Figure 5a,b show the output and transfer characteristics of this device. The G_on_ normalized by the width of the BP channel is 0.12 μS μm^−1^. The device exhibited an on/off current ratio of 12. In contrast, with Joule heating to improve the contact, the device exhibited more than one order of magnitude higher G_on_ per unit channel width and a 404 times higher on/off current ratio, demonstrating the effectiveness of the Joule heating method in enhancing BP-FET performance. The enhancement of the performance of BP-FET with the localized Joule-heating method can be attributed to the formation of Au_2_P_3_, which resulted in the reduced contact resistance and the increased gate regulation efficiency for the device. For the device in Figure 4b, the contact type of the device was changed from the side contact to the end contact due to the formation of the Au_2_P_3_ after the electrical annealing. In contrast, the device in Figure 5b has a side contact between the BP and the metal. According to previous literature [37,38,39], the device with the end contact has a higher gate regulation capacity than the device with the side contact. Therefore, the device in Figure 4b can be turned off better, resulting in a lower off-state current for the device in Figure 4b than that for the device in Figure 5b.

## 4. Conclusions

A localized Joule-heating method was used to improve the contact and performance of BP-FETs with 2D BP on the metal electrodes. The G_0_ of the device increased by approximately five orders of magnitude, and the linearity of the current–voltage curves was also enhanced when a sufficiently large V_ds_ was applied to the device. This improvement in contact can be attributed to the formation of gold phosphide at the interface of the BP and the Au electrodes owing to the current-generated localized Joule heat. The fabricated BP-FET showed a high on/off ratio of 4850 and an on-state conductance per unit channel width of 1.25 μS μm^−1^. This device exhibited a significantly larger on/off current ratio and G_on_ per unit channel width than those of the BP-FETs without the electrical annealing. Overall, this study demonstrated a reliable method for achieving a low-resistance BP/metal contact, enabling the fabrication of high-performance BP-based electronic devices.

## Data Availability

The data presented in this study are available on request from the corresponding author.
